# The End Is the Beginning: Parkinson’s Disease in the Light of Brain Imaging

**DOI:** 10.3389/fnagi.2017.00330

**Published:** 2017-10-10

**Authors:** Arianna Bellucci, Angelo Antonini, Marina Pizzi, PierFranco Spano

**Affiliations:** ^1^Department of Molecular and Translational Medicine, University of Brescia, Brescia, Italy; ^2^Laboratory of Preventive and Personalized Medicine, University of Brescia, Brescia, Italy; ^3^Department of Neurosciences, University of Padova, Padova, Italy; ^4^Istituto Di Ricovero e Cura a Carattere Scientifico (IRCCS) San Camillo, Venezia, Italy

**Keywords:** Parkinson’s disease, synaptic terminal loss, axonal damage, α-synuclein, brain imaging

## Abstract

Parkinson’s disease (PD), the most common neurodegenerative disorder, is characterized by abnormal accumulation of α-synuclein aggregates known as Lewy bodies (LB) and loss of nigrostriatal dopaminergic neurons. Recent neuroimaging studies suggest that in the early phases of PD, synaptic and axonal damage anticipate the onset of a frank neuronal death. Paralleling, even post mortem studies on the brain of affected patients and on animal models support that synapses might represent the primary sites of functional and pathological changes. Indeed, α-synuclein microaggregation and spreading at terminals, by dysregulating the synaptic junction, would block neurotransmitter release, thus triggering a retrograde neurodegenerative process ending with neuronal cell loss by proceeding through the axons. Rather than neurodegeneration, loss of dopaminergic neuronal endings and axons could thus underlie the onset of connectome dysfunction and symptoms in PD and parkinsonisms. However, the manifold biases deriving from the interpretation of human brain imaging data hinder the validation of this hypothesis. Here, we present pivotal evidence supporting that novel comparative brain imaging studies, in patients and experimental models of PD in preliminary stages of disease, could be instrumental for proving whether synaptic endings are the sites where degeneration begins and initiating the factual achievement of disease modifying approaches. The need for such investigations is timely to define an early therapeutic window of intervention to attempt disease halting by terminal and/or axonal healing.

## Perspective

Parkinson’s disease (PD) is the most common neurodegenerative movement disorder. The brain of affected patients is characterized by accumulation of insoluble α-synuclein aggregates in Lewy bodies (LB) and Lewy neurites (LN; Spillantini et al., 1997) and degeneration of the nigrostriatal dopamine system (Surmeier et al., 2017). It has become increasingly evident that in addition to motor, also non-motor symptoms like depression, olfactory dysfunction, constipation and idiopatic REM sleep behavior disorder (IRBD), could precede the onset of movement disturbances in PD by many years (Chaudhuri et al., 2011; Iranzo et al., 2011; Fereshtehnejad et al., 2017).

On this line, evidence supports that PD patients exhibit dysfunctions of multi-neurotransmitter pathways as well as central and peripheral α-synuclein accumulation (Titova et al., 2017). The different functional reserve of affected neuronal networks originating in the central and peripheral nervous system may steer the resilience to symptom onset (Engelender and Isacson, 2017). Prodromal signs would manifest because distinct populations of central and peripheral neurons hold increased predisposition toward α-synuclein accumulation and low functional reserve. Conversely, the onset of motor symptoms originating from nigrostriatal pathology, would be delayed by the larger functional reserve of midbrain dopamine neurons and basal ganglia circuits (Engelender and Isacson, 2017). Therefore, prodromal markers may be identified, opening new hopes for the detection of early pre-degenerative changes that could be instrumental to understand when and where this disorder originates.

It has been described that a series of specific cell-autonomous pathways may significantly contribute to the selective vulnerability of nigrostriatal dopaminergic neurons to α-synuclein accumulation (Surmeier et al., 2017). These include their intrinsic pacemaking activity, Ca^2+^ loading due to sustained voltage-gated Ca^2+^ channel opening, their long and widely diffused axonal arborization and the selective dependency on α-synuclein for synaptic activity. However, the slow progression of disease and the decreasing effect of dopamine substitution therapy over time supports a gradually increasing presynaptic failure that precedes neuronal cell death (Schulz-Schaeffer, 2015). Consistently, recent neuroimaging studies show terminal damage anticipating axonal degeneration, which in turn culminates in dopamine cell loss. Measures of dopamine transporter (DAT) activity in the nigrostriatal and mesolimbic systems in patients in the early phases of PD has unraveled initially more prominent neurodegeneration in dopamine nerve terminals and axons and less severe in the substantia nigra (Caminiti et al., 2017). Magnetic resonance imaging (MRI) studies with diffusion tensor imaging (DTI), a technique allowing detection of microstructural white matter pathology (Tir et al., 2009; Olde Dubbelink et al., 2013; Wang et al., 2017) have led to similar results. In addition, asymmetric nigrostriatal neurodegeneration in early PD mirrors the initial asymmetry in clinical manifestations (Wang et al., 2015). Most strikingly, in IRBD subjects the decrease of striatal DAT binding reflects the rate of progressive dopaminergic dysfunction (Iranzo et al., 2011). The significant reduction in striatal DAT binding, with preserved nigral signal measured with 7T MRI, that we observed in a still asymptomatic family member carrying the Leucine-rich repeat kinase 2 (LRRK2) G2019S mutation, further supports the concept of synaptic dysfunction anticipating Nigrosome-1 degeneration even in familial PD (Ceravolo et al., 2015). Moreover, a study on PD patients with abnormal bilateral DAT binding analyzed by 3 Tesla (3T) MRI revealed that 14 among the 126 PD patients had bilaterally intact Nigrosome-1 and that this parameter was unilaterally unaffected in 7 among the 126 PD patients (Bae et al., 2016).

In line with the idea that early terminal damage mirrors the progression of dopamine denervation rather than cell loss, Saari et al. (2017) have provided evidence suggesting lack of correlation between number of substantia nigra neurons and striatal DAT loss in PD. This further reinforces the concept of reduced DAT binding reflecting axonal dysfunction, abatement of DAT expression or a decrease of its membrane expression rather than the number of viable neurons. Consistently, PD patients in the early phases have been found to display a temporal longitudinal evolution of compensatory changes in the striatum. Indeed, striatal DAT decrease was found to anticipate the loss of vesicular monoamine transporter 2 (VMAT-2) that rapidly disappears and is considered an optimal biomarker to detect nigrostriatal damage in the presymptomatic phases of the disorder (Chen et al., 2008; Nandhagopal et al., 2011). When taken together, these evidences seem particularly consistent with the possibility that in PD degeneration begins at synaptic terminals.

**Figure 1:**
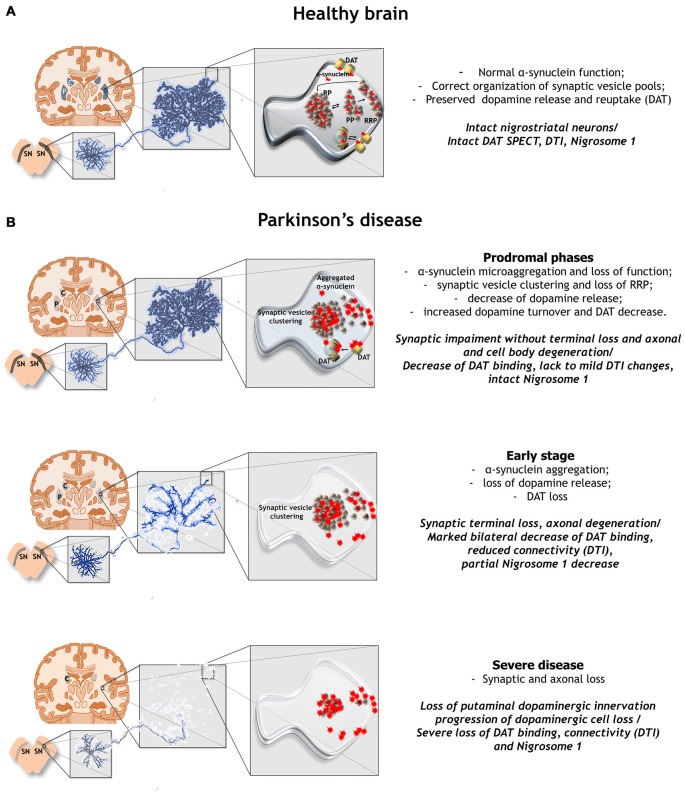
The putative organization of the dopaminergic synapse in healthy condition and in Parkinson’s disease (PD) is presented in relation with nigrostriatal neuron degeneration, connectome function and the disease staging. **(A)** Correct organization of synaptic vesicle pools in a dopaminergic striatal terminal in the healthy brain. **(B)** Retrograde synapse-to-cell-body degeneration initiating and perpetrating nigrostriatal connectome dysfunction in PD. In the prodromal phases of PD, microaggregation of α-synuclein at synaptic terminals progressively changes the organization of synaptic vesicle pools, reduces dopamine release, alters dopamine turnover and decreases dopamine transporter (DAT) membrane content but nigral neuron loss is negligible. Alpha-synuclein can also spread from terminals. DAT binding is reduced with mild to absent alterations of diffusion tensor imaging (DTI) and lack of Nigrosome-1 decrease. This synaptic impairment may initiate connectome dysfunctions in the absence of marked synapse loss or axonal and cell body degeneration. In the early stages of PD, the onset of symptoms is related to the beginning of connectome deficits that mainly arise from synaptic and axonal loss and only to a lesser extent to nigral cell loss. DAT binding and DTI abnormalities are accompanied by a partial or unilateral decrease of Nigrosome-1. Finally, in the advanced phases of the disease, broad synaptic, axonal and cell body degeneration that can be detected by integrating multiple imaging techniques, concomitantly participate to disease progression. PP, Proximal pool; RRP, ready releasable pool, RP, reserve pool.

Studies support that α-synuclein microaggregation at the synapse may be the causative factor initiating dopamine neuron degeneration in PD by impinging on synaptic activity (Schulz-Schaeffer, 2015; Calo et al., 2016). Synaptic α-synuclein microaggregation occurs in the early stages of the degenerative process and in the post-mortem brain of PD patients the load of α-synuclein at terminals is several orders of magnitude higher than its content within LB (Schulz-Schaeffer, 2010). Alpha-synuclein is physiologically enriched at synaptic terminals where it controls neurotransmitter reuptake and homeostasis by regulating transporters as well as synaptic vesicle fusion, clustering, and trafficking between the reserve and ready-releasable pools (Burré, 2015). Alpha-synuclein multimers and aggregates can cluster synaptic vesicles, attenuate their recycling and impair the distribution of associated proteins that are modulated by α-synuclein in physiological conditions (Garcia-Reitböck et al., 2010; Bellucci et al., 2011; Lundblad et al., 2012; Wang et al., 2014; Zaltieri et al., 2015). Among them, Soluble NSF Attachment Protein Receptor (SNARE) proteins, synapsin III, VMAT-2 and the DAT are altered in the nigrostriatal system of experimental models of PD (Garcia-Reitböck et al., 2010; Bellucci et al., 2011; Lundblad et al., 2012; Zaltieri et al., 2015). Notably, mice transgenic for wild type α-synuclein display augmented striatal tonic dopamine release and increased locomotor activity before the onset of striatal denervation and L-DOPA-responsive motor phenotype (Lam et al., 2011), thus supporting that early striatal synaptic dysfunctions precede neurodegeneration. In addition, the early paradoxical increase of dopamine in the olfactory bulb in the absence of motor impairment that has been recently found to occur in 1-Methyl-4-phenyl-1,2,3,6-tetrahydropyridine (MPTP) treated monkeys (Pifl et al., 2017) hints that synaptic changes precede the onset of PD symptoms. Since cell-to-cell transmission of α-synuclein proceeds via trans-synaptic spreading through intact neuronal connections (Ulusoy et al., 2015), it is likely that in the initial phases of PD its deposition at terminals might be the predominant factor prompting even diffusion of pathology (Longhena et al., 2017). Loss of neuronal connections deriving from α-synuclein deposition at terminals could trigger axonal damage, which by proceeding in a retrograde modality, would end with neuronal cell body degeneration (Figure 1). This could occur through the collapse of intracellular trafficking, with the massive axonal harbor of midbrain dopaminergic neurons constituting a pivotal vulnerability factor to intracellular trafficking defects (Hunn et al., 2015).

Therefore, deficits of functional connectivity in the early phases of PD, may underlie the onset of motor and non-motor symptoms and reflect the occurrence of synaptic and axonal degeneration rather than neuronal death. Nonetheless, this still needs to be validated.

The demand of imaging studies aimed at confirming whether neuronal endings are the sites where neurodegeneration begins in PD is thus urgent. In addition, it is timely to define an earlier imaging window. The establishment of novel criteria for the identification of patients in the prodromal phases of disease can offer new opportunities for the establishment of novel comparative brain imaging studies on such subjects and experimental models. These are warranted to provide new insights on when and how nerve terminal degeneration originates and for revising the staging of these disorders. Indeed, when taking into consideration the centrality of nigrostriatal degeneration in the onset of motor symptoms, we could envisage that in the prodromal/early stages of PD there is a phase when striatal synaptic impairment occurs in the absence of axonal degeneration and only subsequently progresses toward nigral cell loss. However, the molecular events involved in this process can only partially be pictured through the integration of data deriving from human brain imaging and studies in experimental models of PD, but we still miss comparative analysis demonstrating how connectome dysfunction originates and progresses. Indeed, pioneering studies have demonstrated a high correlation between positron emission tomography based striatal DAT imaging or DTI and immunostaining values in experimental models of PD such as 6-hydroxydopamine-injected rats and MPTP-treated monkeys (Blesa et al., 2012; Hikishima et al., 2015; Molinet-Dronda et al., 2015), suggesting that comparative analysis is instrumental to decipher live imaging results. This could allow to shed light on the precise molecular alterations underlying single photon emission computed tomography (SPECT), MRI and DTI abnormalities in the early phases of PD. What is more, studies on experimental models of PD should finally benefit from the application of intact brain analysis, which allows to visualize whole nigrostriatal neurons and to discriminate their different conformations (Lerner et al., 2015). This technique could be helpful for evaluating the effective relevance of brain imaging biases deriving from the fact that an enormous amount of synapses derive from a single dopaminergic neuron (Matsuda et al., 2009), as this implies that while the loss of few nigral neurons might be below of the imaging threshold, the loss of the corresponding terminal fields might be easily detectable with imaging. Furthermore, fluorescence lifetime imaging (FLIM), that can detect of α-synuclein conformational changes and oligomers (Outeiro et al., 2009), when coupled with two-photon imaging, could represent an invaluable tool to connect terminal loss with α-synuclein aggregation or to define the pathological “interactome” of the protein that drives neuronal degeneration. Comparative MRI-based studies in animal models undergoing *ex vivo* or intravital microscopy analysis should thus be undertaken to enable a more precise interpretation of data deriving from human brain imaging. These investigations, allowing the correlation between results from human brain imaging and the underlying microscopic neuropathological alterations, could definitely give us novel decoding keys to understand timing and features of connectome dysfunction in the prodromal phases of PD. Hopefully, if brain imaging could allow to determine whether synaptic endings are the sites where PD begins, we may expect to identify a novel therapeutic window of intervention to halt or delay disease progression.

## Author Contributions

AB wrote the first draft; AB, AA, MP and PS prepared the manuscript; managed review and critiques of the manuscript.

## Conflict of Interest Statement

The authors declare that the research was conducted in the absence of any commercial or financial relationships that could be construed as a potential conflict of interest.
